# Tacrolimus Modulates TGF-β Signaling–Related Genes and MicroRNAs in Human Retinal Pigment Epithelial Cells Activated by Lipopolysaccharide

**DOI:** 10.3390/ijms26115402

**Published:** 2025-06-04

**Authors:** Aleksandra Kiełbasińska, Katarzyna Krysik, Dominika Janiszewska-Bil, Martyna Machaj, Zuzanna Lelek, Joanna Sułkowska, Olga Nawotny-Czupryna, Beniamin Oskar Grabarek

**Affiliations:** 1 Department of Opthalmology, University Clinical Center Named After Prof. K. Gibiński of the Medical University of Silesia in Katowice, 40-514 Katowice, Poland; 2 Department of Ophthalmology, St. Barbara Hospital, Trauma Centre, 41-200 Sosnowiec, Poland; kkrysik@gmail.com (K.K.); dominikabjaniszewska@gmail.com (D.J.-B.); martyna.a.machaj@gmail.com (M.M.); zuzkalelek@gmail.com (Z.L.); 3 Department of Ophthalmology, Faculty of Medicine, Academy of Silesia, 40-555 Katowice, Poland; 4 Collegium Medicum, WSB University, 41-300 Dabrowa Gornicza, Poland; joanna.sulkowska@wsb.edu.pl (J.S.); onowotny-czupryna@wsb.edu.pl (O.N.-C.); bgrabarek7@gmail.com (B.O.G.)

**Keywords:** retinal pigment epithelium, transforming growth factor-beta (TGF-β) signaling pathway, proliferative vitreoretinopathy, tacrolimus, lipopolysaccharide (LPS)

## Abstract

The retinal pigment epithelium (RPE) plays a crucial role in maintaining retinal homeostasis, and dysregulation of the transforming growth factor-beta (TGF-β) signaling pathways contributes to retinal fibrosis and inflammatory diseases, including proliferative vitreoretinopathy (PVR). Tacrolimus (FK506), an immunosuppressant, has shown potential antifibrotic properties, but its effects on TGF-β-related genes and microRNAs (miRNAs) in RPE cells remain unclear. Human RPE (H-RPE) cells were treated with lipopolysaccharide (LPS) to induce inflammation and subsequently exposed to tacrolimus. Gene and miRNA expression profiling related to TGF-β signaling pathways were conducted using microarrays, followed by Quantitative Reverse-Transcription Polymerase Chain Reaction (RT-qPCR) validation. Protein levels were assessed via enzyme-linked immunosorbent assay (ELISA), and interactions were analyzed using STRING database network analysis. Tacrolimus modulated key components of the TGF-β pathway, upregulating TGF-β2, TGF-β3, SMAD2, and SMAD4 while downregulating TGF-βR1 and SMAD7. JAK/STAT and MAPK pathways were also affected, indicating broad regulatory effects. miRNA profiling identified hsa-miR-200a-3p, hsa-miR-589-3p, hsa-miR-21, and hsa-miR-27a-5p as key regulators. STRING analysis confirmed strong functional interactions within the TGF-β network. In conclusion, tacrolimus modulates both canonical (upregulation of SMAD2/4 and downregulation of SMAD7) and non-canonical (JAK/STAT and MAPK) TGF-β signaling pathways in LPS-stimulated RPE cells. These changes collectively suggest a dual anti-inflammatory and anti-fibrotic effect. The increased TGF-β2 and decreased SMAD7 levels, alongside altered miRNA expression (e.g., downregulation of miR-200a-3p), indicate that tacrolimus may inhibit key profibrotic mechanisms underlying PVR. These findings support the potential therapeutic repurposing of tacrolimus in PVR and warrant further in vivo validation.

## 1. Introduction

The retinal pigment epithelium (RPE) plays a crucial role in maintaining retinal homeostasis by supporting photoreceptor survival, forming the blood–retina barrier, and modulating immune responses [1,2]. However, its dysregulation is implicated in several retinal diseases, including age-related macular degeneration (AMD), proliferative vitreoretinopathy (PVR), and diabetic retinopathy (DR), all of which involve chronic inflammation, oxidative stress, and fibrosis [3]. PVR is a major cause of retinal detachment surgery failure, characterized by excessive fibrocellular membrane formation, contraction, and scar tissue development, ultimately leading to vision loss [4].

PVR develops as a consequence of rhegmatogenous retinal detachment (RRD) and represents one of the most common complications of its surgical treatment. RRD occurs due to a full-thickness break in the continuity of the neurosensory retina, allowing vitreous humor to enter the space between the retina and the retinal pigment epithelium (RPE). Most retinal tears, in turn, result from traction caused by posterior vitreous detachment (PVD). PVR develops in approximately 5–10% of RRD cases. If untreated, RRD can lead to PVR within a few weeks to several months [5,6].

PVR is a wound-healing-like process that clinically resembles an aberrant fibrotic response. It is characterized by increased proliferation of RPE cells, glial cells, fibroblasts, and inflammatory cells [7]. Additionally, RPE cells undergo epithelial–mesenchymal transition (EMT), resulting in their transformation into cells with a myofibroblast-like phenotype. This leads to the formation of fibrotic membranes on the inner and outer surfaces of the retina as well as on the surface of the vitreous body. These membranes contract, causing the formation of permanent retinal folds, equatorial traction, detachment of the non-pigmented epithelium of the pars plana of the ciliary body, and generalized retinal shrinkage [8,9,10,11].

The pathogenesis of PVR is not fully understood. It is believed to occur as a repair process triggered by retinal injury or inflammation. Pro-inflammatory cytokines such as interleukin (IL)-1β, IL-2, IL-6, IL-8, and tumor necrosis factor-alpha (TNF-α), along with growth factors such as transforming growth factor-beta (TGF-β), platelet-derived growth factor (PDGF), hepatocyte growth factor (HGF), connective tissue growth factor (CTGF), fibroblast growth factor (FGF), and epidermal growth factor (EGF) play critical roles in the development of PVR. These factors are present in the vitreous humor and are also secreted by RPE cells. Furthermore, glial cells and cells that form PVR membranes release some of these cytokines and growth factors. Additionally, the vitreous contains other biologically active substances, such as proteases and protease inhibitors, which are also involved in the pathogenesis of PVR [6,12,13].

A key driver of these pathological processes is the TGF-β signaling pathway, which orchestrates cellular differentiation, proliferation, and immune regulation [14]. The pathogenesis of PVR involves chronic inflammation, and aberrant activation of TGF-β signaling, making it a critical target for therapeutic intervention [15,16].

The three major isoforms of TGF-β (TGF-β1, TGF-β2, and TGF-β3) have distinct but overlapping roles in retinal health and disease: while TGF-β1 and TGF-β2 are predominantly involved in fibrosis and immune suppression, TGF-β3 has been suggested to have antifibrotic properties under certain conditions [17,18]. Importantly, aberrant activation of the TGF-β pathway in RPE cells promotes EMT, a process that contributes to fibrosis by inducing a shift from an epithelial to a mesenchymal phenotype, characterized by enhanced migration, extracellular matrix deposition, and loss of epithelial junctions [10,19]. This pathological transformation is mediated through both canonical (SMAD-dependent) and non-canonical (SMAD-independent) pathways, where the former involves SMAD2/3 phosphorylation and nuclear translocation, while the latter engages signaling cascades such as MAPK, PI3K/AKT, and Rho/ROCK that further drive inflammation and fibrosis [20,21]. Given the central role of TGF-β in these pathological processes, modulating its activity presents a promising therapeutic strategy [20,21]. Tacrolimus (FK506), a potent immunosuppressant widely used in transplantation medicine, exerts its effects primarily by binding to the FK506-binding protein 12 (FKBP12), thereby inhibiting calcineurin, a phosphatase that dephosphorylates nuclear factor of activated T cells (NFAT), a transcription factor crucial for T-cell activation and cytokine production [22,23]. Beyond its well-established immunosuppressive function, recent studies suggest that tacrolimus may also exert antifibrotic effects by interfering with TGF-β signaling, though the precise mechanisms remain unclear [14]. Notably, tacrolimus has been reported to modulate SMAD signaling, reduce pro-fibrotic gene expression, and inhibit EMT in various cell types, raising the possibility that it could attenuate pathological changes in RPE cells exposed to inflammatory stimuli [24]. Given that lipopolysaccharide (LPS), a component of Gram-negative bacterial cell walls, is known to elicit a strong inflammatory response in RPE cells, it serves as a well-established model for studying retinal inflammation [25,26]. However, the impact of tacrolimus on the expression profile of genes and microRNAs (miRNAs) related to TGF-β signaling pathways in RPE cells under inflammatory conditions remains largely unexplored.

MicroRNAs (miRNAs) are small, non-coding RNAs that play a crucial role in post-transcriptional gene regulation by binding to target messenger RNAs (mRNAs) and either promoting their degradation or inhibiting their translation [27,28]. In the context of TGF-β signaling and retinal diseases, several miRNAs have been identified as key regulators of fibrosis, inflammation, and EMT in RPE cells [29]. For instance, miR-21 is known to promote fibrosis by enhancing SMAD signaling, whereas miR-29 and miR-200 families exhibit antifibrotic properties by inhibiting extracellular matrix deposition and EMT progression [30,31].

Therefore, this study aims to investigate whether tacrolimus modulates the expression of key TGF-β-related genes and miRNAs in LPS-stimulated H-RPE cells, providing novel insights into its potential as a therapeutic agent for inflammatory and fibrotic retinal diseases.

## 2. Results

### 2.1. Results of MTT Cytotoxicity Assay

To determine the optimal experimental conditions for subsequent gene and protein expression analyses, we first performed an MTT cytotoxicity assay using H-RPE cells treated with LPS and tacrolimus across various concentrations and time points (6, 12, and 24 h).

LPS at 1 µg/mL did not significantly affect cell viability at any time point (*p* = 0.784), indicating this concentration was well-tolerated. In contrast, LPS at 2 µg/mL and 10 µg/mL caused statistically significant reductions in cell viability at longer exposure times (*p* = 0.023 and *p* = 0.022, respectively), particularly between 6 and 24 h.

**Figure 1:**
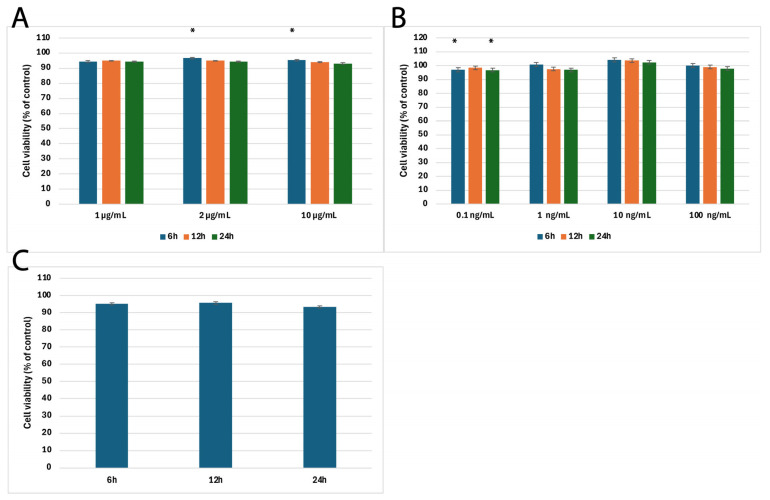
Results of cytotoxicity assay (**A**) Cell viability after exposure to increasing concentrations of LPS (1, 2, 10 µg/mL) at 6, 12, and 24 h; (**B**) Cell viability after exposure to tacrolimus (0.1, 1, 10, 100 ng/mL) at 6, 12, and 24 h; (**C**) Cell viability following sequential treatment: LPS (1 µg/mL for 6 h) followed by tacrolimus (0.1–100 ng/mL) for an additional 6, 12, or 24 h. Data are presented as mean ± SD; n = three independent experiments. Viability was assessed as a percentage of control (untreated) cells. * *p* < 0.05 vs. control at the same time point (one-way ANOVA with Scheffé’s post hoc test); LPS, lipopolysaccharide.

For tacrolimus, a concentration of 0.1 ng/mL significantly affected viability between 6 and 12 h and between 12 and 24 h (*p* = 0.027). However, higher concentrations—1, 10, and 100 ng/mL—did not lead to statistically significant cytotoxicity (*p*-values of 0.077, 0.073, and 0.052, respectively). When LPS and tacrolimus were used in combination, the difference in viability was not statistically significant (*p* = 0.0786), supporting the safety of combined treatment. Based on these results and supporting literature [14,32,33,34,35,36], 1 µg/mL LPS and 10 ng/mL tacrolimus were selected for further experiments. H-RPE cells were first exposed to LPS for 6 h to initiate an inflammatory response, after which tacrolimus was added. This protocol allowed us to investigate the modulatory effects of tacrolimus on an already activated inflammatory state while avoiding cytotoxicity. Figure 1 shows cytotoxicity assay results.

### 2.2. Differential Microarray Expression of TGF-β-Related Genes in H-RPE Cells Treated with LPS, Tacrolimus, and Their Combination

Among the 6151 genes associated with the transforming growth factor-beta (TGF-β) signaling pathways, ANOVA analysis revealed that 4087 genes exhibited statistically significant expression differences in H-RPE cells treated with lipopolysaccharide (LPS), tacrolimus, or their combination compared to control (*p* < 0.05). A subset of 365 genes showed substantial regulation with a fold change (|FC|) > 4.00 in at least one comparison.

Post hoc Tukey analysis identified 176 differentially expressed mRNAs in cells exposed to the combination of LPS and tacrolimus after 6 h, 122 after 12 h, and 87 after 24 h. Further temporal profiling revealed that 80 mRNAs were uniquely altered after 6 h exposure, 19 after 12 h, and 6 after 24 h, while 20 mRNAs were consistently deregulated at all time points. These consistently modulated transcripts included the following: transforming growth factor beta 2 (*TGF-β2*), transforming growth factor beta 3 (*TGF-β3*), transforming growth factor beta receptor 1 (*TGF-βRI*), transforming growth factor beta receptor 3 (*TGF-βRIII*), SMAD family member 2 (*SMAD2*), SMAD family member 4 (*SMAD4*), mitogen-activated protein kinase 3 (MAPK3), Janus kinase 1 (*JAK1*), Janus kinase 2 (*JAK2*), Janus kinase 3 (*JAK3*), signal transducer and activator of transcription 3 (*STAT3*), epidermal growth factor receptor (*EGFR*), SMAD family member 7 (*SMAD7*), interferon gamma (*IFNG*), interleukin 6 (*IL6*), caspase 3 (*CASP3*), interleukin 2 (*IL2*), caspase 8 (*CASP8*), kinase insert domain receptor (*KDR/VEGFR-2*), and prosaposin (*PSAP*). Importantly, gene expression profiling also revealed that tacrolimus alone, even in the absence of prior inflammatory stimulation, significantly modulated the transcription of several key genes in the TGF-β pathway. These included strong upregulation of pro-regulatory and inflammatory markers such as *IL-6*, *STAT3*, *MAPK3*, *IFNG*, and *TGF-β3*, and suppression of inhibitory components like *SMAD7*, *TGF-βR1*, and *CASP3*. These effects suggest that tacrolimus exerts a direct modulatory role on TGF-β signaling in retinal pigment epithelial cells, independent of LPS-induced inflammation.

**Figure 2:**
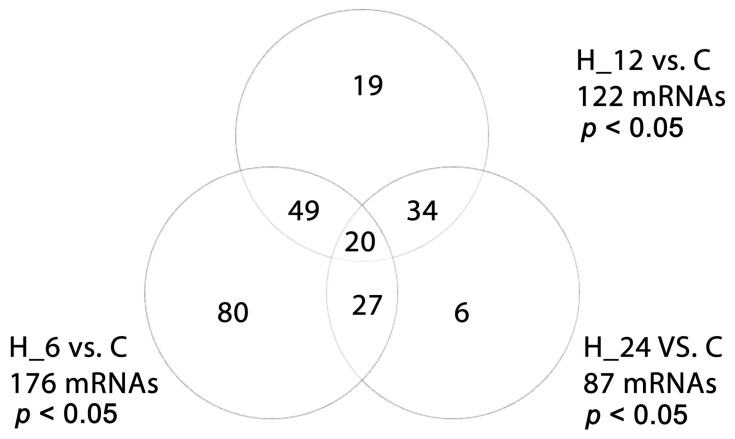
Venn diagram illustrating time-dependent differential expression of TGF-β-related genes in H-RPE cells treated with LPS and tacrolimus. C, control culture; H_6, H_12, H_24, culture exposed to tacrolimus for 6, 12, 24 h.

Figure 2 presents a Venn diagram summarizing time-dependent gene expression overlaps across treatment durations, while Table 1 details the expression dynamics (log_2_ fold change) of 25 representative TGF-β-dependent genes across experimental conditions, including LPS alone, tacrolimus alone, and LPS followed by tacrolimus at different time points.

#### 2.2.1. Changes of Selected Genes Related to TGF-β-Family in H-RPE Cells Treated with LPS or Tacrolimus via RT-qPCR

RT-qPCR validation confirmed distinct transcriptional responses of TGF-β-related genes in H-RPE cells exposed to either LPS or tacrolimus. Exposure to LPS resulted in moderate upregulation of *TGF-β2*, *TGF-β3*, *MAPK3*, *JAK1*, *JAK3*, *STAT3*, and *CASP3*, while key signaling components including *TGF-βR1*, *SMAD2*, *SMAD4*, *JAK2*, *SMAD7*, *EGFR*, *IFNG*, *IL-6*, *KDR*, and *PSAP* were notably downregulated.

**Figure 3:**
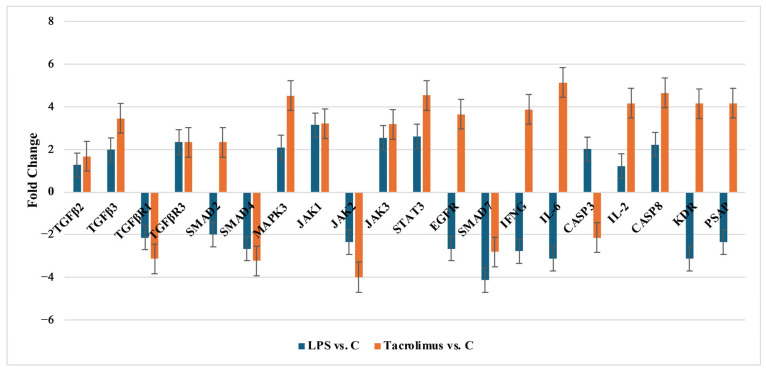
Expression of 20 mRNA in the H-RPE treated with LPS or tacrolimus (RT-qPCR results) presented as fold change in expression when compared to a control culture. Data are presented as mean ± SD; *TGF-β2*, Transforming Growth Factor Beta 2; *TGF-β3*, Transforming Growth Factor Beta 3; *TGF-βR1*, Transforming Growth Factor Beta Receptor 1; *TGF-βR3*, Transforming Growth Factor Beta Receptor 3; *SMAD2*, Mothers Against Decapentaplegic Homolog 2; *SMAD4*, Mothers Against Decapentaplegic Homolog 4; *MAPK3*, Mitogen-Activated Protein Kinase 3 (ERK1); *JAK1*, Janus Kinase 1; *JAK2*, Janus Kinase 2; *JAK3*, Janus Kinase 3; *STAT3*, Signal Transducer and Activator of Transcription 3; *EGFR*, Epidermal Growth Factor Receptor; *SMAD7*, Mothers Against Decapentaplegic Homolog 7; *IFNG*, Interferon Gamma; *IL-6*, Interleukin 6; *CASP3*, Caspase 3; *IL-2*, Interleukin 2; *CASP8*, Caspase 8; *KDR*, Kinase Insert Domain Receptor (Vascular Endothelial Growth Factor Receptor 2, VEGFR-2); *PSAP*, Prosaposin.

In contrast, tacrolimus alone significantly induced the expression of pro-regenerative and immune-regulatory genes such as *TGF-β2*, *TGF-β3*, *MAPK3*, *STAT3*, *IL-6*, *IL-2*, *CASP8*, *EGFR*, and *KDR*. Concurrently, it strongly suppressed *TGF-βR1*, *SMAD4*, *JAK2*, *SMAD7*, and *CASP3*, indicating a broader immunomodulatory effect even in the absence of an inflammatory stimulus. Figure 3 presents RTq-PCR results. Statistical significance (*p* < 0.05) was determined by *t*-Student’s test, which are summarized in Table A1.

#### 2.2.2. Changes of Selected Genes Related to TGF-β-Family in H-RPE Cells Treated with LPS and Tacrolimus via RT-qPCR

**Figure 4:**
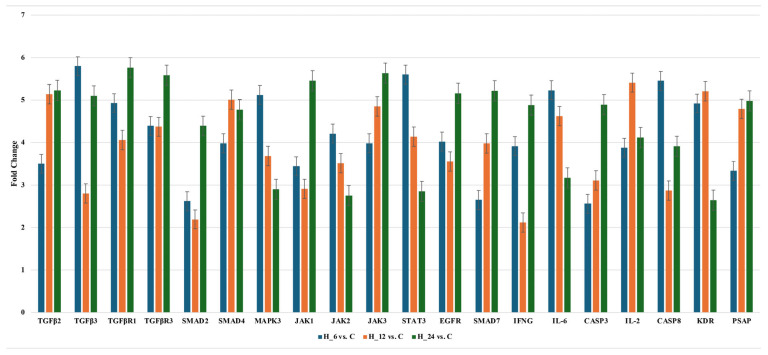
Expression of 20 mRNA in the H-RPE treated with LPS and tacrolimus (RT-qPCR results) presented as fold change in expression when compared to a control culture. Data are presented as mean ± SD; *TGF-β2*, Transforming Growth Factor Beta 2; *TGF-β3*, Transforming Growth Factor Beta 3; *TGF-βR1*, Transforming Growth Factor Beta Receptor 1; *TGF-βR3*, Transforming Growth Factor Beta Receptor 3; *SMAD2*, Mothers Against Decapentaplegic Homolog 2; *SMAD4*, Mothers Against Decapentaplegic Homolog 4; *MAPK3*, Mitogen-Activated Protein Kinase 3 (ERK1); *JAK1*, Janus Kinase 1; *JAK2*, Janus Kinase 2; *JAK3*, Janus Kinase 3; *STAT3*, Signal Transducer and Activator of Transcription 3; *EGFR*, Epidermal Growth Factor Receptor; *SMAD7*, Mothers Against Decapentaplegic Homolog 7; *IFNG*, Interferon Gamma; *IL-6*, Interleukin 6; *CASP3*, Caspase 3; *IL-2*, Interleukin 2; *CASP8*, Caspase 8; *KDR*, Kinase Insert Domain Receptor (Vascular Endothelial Growth Factor Receptor 2, VEGFR-2); *PSAP*, Prosaposin.

The data indicate dynamic changes in gene expression over time. *TGF-β2*, *TGF-β3*, and *TGF-βR3* are upregulated, while *TGF-βR1* is downregulated. *SMAD2* and *SMAD4* show increased expression, whereas *SMAD7* is downregulated. *JAK1* and *STAT3* are upregulated, while *JAK2* and *JAK3* are downregulated. *EGFR* expression remains elevated. *MAPK3*, *IFNG*, and *IL6* are consistently downregulated. Apoptotic markers *CASP3* and *CASP8* are upregulated, and *IL-2* expression fluctuates. *KDR* shows an early peak, while *PSAP* is downregulated at all time points. Figure 4 presents RTq-PCR results. Statistical significance was determined by ANOVA with post hoc Scheffé’s test. Statistically significant differences (*p* < 0.05) were summarized in Table A2.

### 2.3. Differential miRNA Expression in H-RPE Cells Treated with LPS, Tacrolimus, or Their Combination

Microarray analysis was performed to assess changes in miRNA expression in H-RPE cells treated with LPS, tacrolimus, or their combination. The analysis focused on miRNAs with high target prediction scores (Target score > 90) that are likely to regulate genes within the TGF-β signaling pathway. The results revealed distinct, condition-specific expression patterns suggesting post-transcriptional regulation of key mRNAs.

hsa-miR-200a-3p, targeting *TGF-β2*, was significantly upregulated in response to LPS (+1.98 ± 0.76) and tacrolimus (+1.65 ± 0.61) alone (*p* < 0.05), but markedly downregulated following combined LPS + tacrolimus treatment at 6, 12, and 24 h (−2.54 to −2.11, *p* < 0.05), indicating treatment-specific, biphasic modulation of this miRNA and its regulatory impact on TGF-β2 expression.

hsa-miR-589-3p, a strong predicted regulator of *SMAD4*, showed significant downregulation after LPS (−2.12 ± 0.34) and tacrolimus (−2.98 ± 0.61) exposure alone (*p* < 0.05), but was strongly upregulated in all LPS + tacrolimus conditions (+3.21 to +2.16, *p* < 0.05), consistent with temporal post-transcriptional derepression of SMAD4 during combined treatment.

hsa-miR-21, targeting SMAD7, was upregulated in all groups, with increasing expression under tacrolimus alone (+1.98 ± 0.31, *p* < 0.05) and LPS + tacrolimus (+1.87 to +2.02, *p* < 0.05), while LPS alone induced a modest increase (+1.44 ± 0.17, *p* < 0.05). This may contribute to the fine-tuning of SMAD7-dependent inhibitory signaling within the TGF-β cascade.

hsa-miR-27a-5p, predicted to regulate IL-2, was upregulated by both LPS (+2.09 ± 0.65) and tacrolimus (+2.12 ± 0.19) treatments (*p* < 0.05), but strongly downregulated under LPS + tacrolimus exposure (−2.13 to −2.11, *p* < 0.05). These findings suggest opposite regulatory trends when treatments are combined versus applied individually.

Detailed results for all experimental conditions and time points are presented in Table 2.

### 2.4. Results of Concentration of Selected Proteins Enzyme-Linked Immunosorbent Assay (ELISA Assay)

Protein concentrations of selected components involved in TGF-β signaling were evaluated by enzyme-linked immunosorbent assay (ELISA) in H-RPE cells treated with LPS, tacrolimus, or their combination.

TGF-β2 protein levels increased significantly across all treatments. A modest rise was observed following LPS alone (328.76 ± 8.91 pg/mL) and tacrolimus alone (182.34 ± 5.22 pg/mL) compared to the control (124.11 ± 3.45 pg/mL). The most pronounced increase occurred in cells exposed to LPS followed by tacrolimus, with levels rising to 561.98 ± 9.12 pg/mL at 6 h and peaking at 716.11 ± 6.71 pg/mL at 24 h (*p* < 0.05).

SMAD4 concentrations, in contrast, decreased steadily over time under the combination treatment—from 3.45 ± 0.12 ng/mL at 6 h to 2.99 ± 0.19 ng/mL at 24 h—despite initial increases in LPS (6.12 ± 0.44 ng/mL) and tacrolimus-only (6.89 ± 0.51 ng/mL) conditions. This inverse pattern may reflect complex transcriptional and post-transcriptional regulation involving miR-589-3p.

SMAD7 protein levels also declined in all experimental groups. Compared to the control (6.19 ± 0.65 ng/mL), levels were significantly lower in the LPS (5.10 ± 0.35 ng/mL) and tacrolimus (4.58 ± 0.42 ng/mL) groups, with further reductions following combined treatment (3.45 ± 0.19 ng/mL at 24 h, *p* < 0.05), supporting the role of miR-21 in post-transcriptional suppression.

IL-2 showed a distinct pattern. While LPS (122.11 ± 9.12 pg/mL) and tacrolimus (98.76 ± 8.45 pg/mL) alone reduced protein levels relative to control (176.87 ± 12.65 pg/mL), combined treatment resulted in a robust upregulation, with levels reaching 245.17 ± 19.91 pg/mL at 6 h and increasing to 671.91 ± 13.45 pg/mL at 24 h (*p* < 0.05), consistent with miR-27a-5p suppression in the combination group.

The complete quantitative data are presented in Table 3.

### 2.5. STRING Analysis

**Figure 5:**
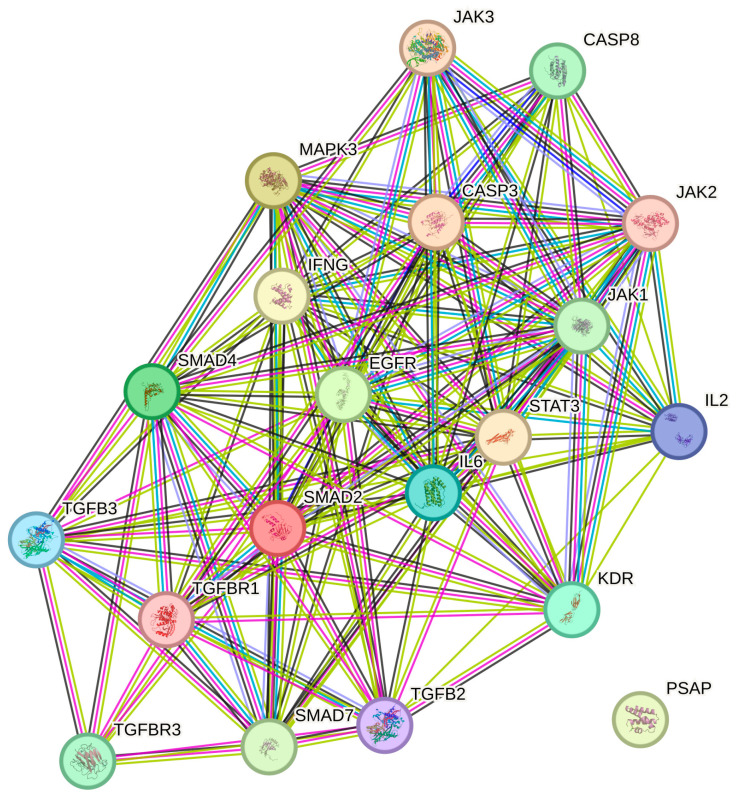
Protein interaction network for TGF- β system differentiation-related genes generated using the STRING database. *TGF-β2*, Transforming Growth Factor Beta 2; *TGF-β3*, Transforming Growth Factor Beta 3; *TGF-βR1*, Transforming Growth Factor Beta Receptor 1; *TGF-βR3*, Transforming Growth Factor Beta Receptor 3; *SMAD2*, Mothers Against Decapentaplegic Homolog 2; *SMAD4*, Mothers Against Decapentaplegic Homolog 4; *MAPK3*, Mitogen-Activated Protein Kinase 3 (ERK1); *JAK1*, Janus Kinase 1; *JAK2*, Janus Kinase 2; *JAK3*, Janus Kinase 3; *STAT3*, Signal Transducer and Activator of Transcription 3; *EGFR*, Epidermal Growth Factor Receptor; *SMAD7*, Mothers Against Decapentaplegic Homolog 7; *IFNG*, Interferon Gamma; *IL-6*, Interleukin 6; *CASP3*, Caspase 3; *IL-2*, Interleukin 2; *CASP8*, Caspase 8; *KDR*, Kinase Insert Domain Receptor (Vascular Endothelial Growth Factor Receptor 2, VEGFR-2); *PSAP*, Prosaposin.

The STRING database analysis reveals a highly interconnected protein–protein interaction (PPI) network of 20 nodes and 133 edges, significantly exceeding the expected 38 edges (PPI enrichment *p*-value < 1 × 10^−16^). The average node degree of 13.3 indicates strong connectivity between genes, while the high local clustering coefficient (0.815) suggests well-organized functional interactions. These findings highlight significant enrichment and strong associations within the analyzed histaminergic system differentiation-related genes (Figure 5).

The analyzed gene network is highly interconnected, playing a crucial role in growth factor signaling, immune modulation, and apoptosis regulation. Key enriched biological processes include enzyme-linked and cell surface receptor signaling pathways, cellular response to organic substances, and regulation of cell proliferation (FDR < 1.22 × 10^−14^). Functionally, the network is dominated by cytokine receptor binding, signaling receptor binding, and TGF-beta receptor interactions, highlighting its involvement in inflammatory and growth factor-mediated responses. Cellular component analysis indicates membrane raft and receptor complex localization, suggesting active signal transduction and receptor internalization. The high PPI enrichment (*p* < 1 × 10^−16^) confirms significant non-random interactions, emphasizing the coordinated function of TGF-beta, JAK/STAT, and caspase pathways in cellular regulation.

## 3. Discussion

To further clarify the role of tacrolimus in modulating TGF-β signaling under inflammatory conditions, we extended our analysis to include four distinct experimental groups: untreated control, LPS-only, tacrolimus-only, and LPS followed by tacrolimus. This approach allowed us to distinguish the specific and combined effects of inflammation and immunomodulation. Notably, several key genes such as TGF-β2, SMAD4, and STAT3 were significantly regulated not only in the combined treatment but also independently by LPS or tacrolimus. For instance, tacrolimus alone increased the expression of TGF-β2 and IL-6, while downregulating SMAD7, suggesting that its immunomodulatory and antifibrotic effects are not solely dependent on pre-existing inflammatory stimuli. Conversely, the LPS-only group showed distinct patterns of gene activation reflective of a pro-inflammatory state. The combined LPS + tacrolimus treatment induced synergistic or counter-regulatory expression profiles for several components of the TGF-β signaling cascade and related miRNAs. These findings underscore the necessity of including both single-agent and combination groups in studies evaluating therapeutic interventions, as it allows for a more accurate delineation of drug-specific effects versus those arising from interaction with inflammatory processes. This experimental design strengthens the translational relevance of our findings and provides a mechanistic rationale for the potential use of tacrolimus in modulating retinal fibrosis and inflammation.

Our transcriptomic and proteomic analyses revealed distinct regulatory effects of tacrolimus on key components of the TGF-β pathways.

The results demonstrated an upregulation of *TGF-β2* and *TGF-β3*, accompanied by a notable downregulation of *TGF-βR1*. This suggests that tacrolimus may alter the balance of TGF-β isoforms, potentially favoring antifibrotic responses [35,37]. Prior studies have established that *TGF-β1* and *TGF-β2* drive fibrosis in various tissues, including the retina, whereas *TGF-β3* has been implicated in antifibrotic mechanisms [38]. The suppression of TGF-βR1 may limit canonical SMAD-dependent signaling [39], potentially attenuating the profibrotic responses often associated with PVR and other retinal fibrotic diseases [40,41].

Interestingly, the concurrent upregulation of *SMAD2* and *SMAD4* and downregulation of *SMAD7* suggests an enhancement of canonical TGF-β signaling [42,43]. SMAD7 is a well-established inhibitory SMAD, preventing SMAD2/3 activation and thereby negatively regulating fibrosis [44]. The decrease in *SMAD7* levels suggests that tacrolimus may facilitate controlled TGF-β signaling activation, potentially to modulate immune responses rather than promoting fibrosis [45,46,47]. Previous studies in fibrotic lung and kidney models have similarly reported tacrolimus-induced modulation of the SMAD pathway [24]. Given that PVR involves excessive TGF-β-driven fibrosis, targeting these pathways could offer new therapeutic avenues.

Beyond the canonical SMAD-dependent pathway, our results indicate that tacrolimus exerts significant influence over non-canonical TGF-β signaling components, particularly the JAK/STAT [48] and MAPK pathways [21]. The observed upregulation of *JAK1*, *JAK3*, and *STAT3*, combined with downregulation of *JAK2*, suggests that tacrolimus may selectively regulate these signaling networks. The JAK/STAT pathway is integral to immune response regulation, and its selective modulation could provide anti-inflammatory benefits while preserving cellular homeostasis [48,49].

The concurrent downregulation of EGFR and upregulation of MAPK3 further suggests that tacrolimus regulates proliferative signals in a time-dependent manner [50,51]. EGFR inhibition has been linked to reduced fibrotic remodeling in retinal disorders, while MAPK3 signaling plays a context-dependent role in cell survival and differentiation [20]. These findings indicate that tacrolimus does not uniformly suppress all non-canonical pathways but rather fine-tunes their activity, potentially providing a dual benefit: inhibiting fibrosis while maintaining necessary repair mechanisms.

Our study identified several miRNA-gene interactions that contribute to the regulatory effects of tacrolimus. Notably, hsa-miR-200a-3p was downregulated, leading to increased TGF-β2 expression, while hsa-miR-589-3p upregulated SMAD4. These findings align with prior research showing that miR-200a inhibits EMT and fibrosis by suppressing TGF-β signaling [29,30].

The upregulation of hsa-miR-21, a known positive regulator of SMAD7, raises an interesting contradiction, as SMAD7 was downregulated at the mRNA and protein levels [52,53,54]. This suggests that post-transcriptional modifications or feedback inhibition mechanisms may be involved [55,56]. The observed suppression of hsa-miR-27a-5p, which negatively regulates IL-2, supports the notion that tacrolimus promotes immunostimulatory effects while maintaining a degree of immune suppression through calcineurin inhibition [57]. These findings highlight miRNA-based mechanisms as potential therapeutic targets in PVR and other fibrotic conditions [58,59]. Additionally, the ELISA results confirmed transcriptional changes, with a marked increase in TGF-β2 and IL-2 levels, alongside significant reductions in SMAD4 and SMAD7 concentrations. Notably, the increase in IL-2 levels suggests that tacrolimus enhances immune activity, which could contribute to immune surveillance and tissue repair mechanisms in the retina [45,46,47]. This dual role of tacrolimus—modulating immune responses while controlling fibrosis—could be particularly valuable in treating PVR, where excessive immune activation often leads to recurrent retinal detachment [60].

In turn, the STRING network analysis further validated these molecular interactions, revealing a highly interconnected PPI network with significant enrichment in cytokine receptor binding, growth factor signaling, and apoptosis regulation (PPI enrichment *p* < 1 × 10^−16^). These findings reinforce the functional relevance of the identified molecular changes, highlighting the complex interplay between immune modulation, fibrosis suppression, and cell survival.

Given that PVR remains a major challenge in vitreoretinal surgery, often leading to vision loss due to excessive fibrosis [60] and recurrent detachment, these findings suggest that tacrolimus could be repurposed as an anti-fibrotic and immunomodulatory agent. Its ability to modulate TGF-β and JAK/STAT signaling may provide dual benefits—attenuating fibrotic remodeling while preserving essential immune responses [45,46,47].

While our study provides a comprehensive transcriptomic and miRNA-level analysis of TGF-β signaling modulation by tacrolimus in an inflammatory RPE model, it is important to acknowledge that interpretation of signaling pathway activity based solely on mRNA expression has inherent limitations. Although we partially addressed this through protein quantification using ELISA for selected targets (TGF-β2, SMAD4, SMAD7, and IL-2), the absence of broader protein-level validation and mechanistic interrogation (e.g., via gene knockdown or pathway inhibition) restricts our ability to definitively confirm functional pathway activation or inhibition. Future studies employing techniques such as Western blotting, siRNA-mediated gene silencing, or CRISPR/Cas9-based gene editing are warranted to elucidate the causal relationships between gene expression changes and downstream cellular phenotypes. These approaches will be crucial to validate the regulatory roles of identified targets and strengthen the translational potential of tacrolimus in retinal fibrotic disorders.

## 4. Materials and Methods

### 4.1. Study Design

This study was designed to investigate the modulatory effects of tacrolimus on the expression of genes and microRNAs associated with the TGF-β signaling pathway in an in vitro model of retinal inflammation using H-RPE cells. To simulate inflammatory conditions, H-RPE cells were stimulated with LPS at a concentration of 1 µg/mL. The experimental conditions included untreated control cells, cells treated with LPS alone for 6 h, and cells treated with tacrolimus alone at a concentration of 10 ng/mL for 6 h. Additionally, a combination treatment group was included, in which cells were first incubated with LPS (1 µg/mL) for 6 h to induce a pro-inflammatory state, followed by exposure to tacrolimus (10 ng/mL) for an additional 6, 12, or 24 h.

This sequential treatment protocol was designed to mimic an inflammatory environment followed by immunomodulatory intervention. The gene expression profiles under these various conditions were evaluated using high-density oligonucleotide microarray analysis (HG-U133_A2, Affymetrix, Santa Clara, CA, USA), and microRNA expression was assessed using the GeneChip™ miRNA 2.0 array (Affymetrix, Santa Clara, CA, USA). Selected mRNAs and proteins involved in the TGF-β pathway were further validated by reverse-transcription quantitative PCR (RT-qPCR) and enzyme-linked immunosorbent assay (ELISA), respectively. The cytotoxic effects of LPS and tacrolimus, both individually and in combination, were assessed using the citotoxicity assay to determine appropriate experimental concentrations and exposure durations for the molecular analyses.

### 4.2. Cell Culture and Cytotoxicity Assay

Human retinal pigment epithelial (H-RPE) cells (194987, Clonetics, San Diego, CA, USA) were cultured in 25 cm^2^ cell culture flasks with a Nunclon-coated surface (Nunc, Wiesbaden, Germany). The cells were maintained in retinal pigment epithelial basal medium (RtEBM™; Lonza, Basel, Switzerland) supplemented with fibroblast growth factor-basic (FGF-B) and 2% fetal bovine serum (FBS) (Lonza, Basel, Switzerland) for 24 h post-passage. Cultures were incubated in a Direct Heat CO_2_ Incubator (Thermo Fisher, Waltham, MA, USA).

To induce an inflammatory response, the exposure time and concentration of lipopolysaccharide (LPS; Sigma-Aldrich, Poznań, Poland) were determined experimentally [14,61,62]. H-RPE cells were incubated with LPS at concentrations of 1, 2, and 10 µg/mL in culture medium for 6, 12, and 24 h. Untreated cells served as the control group.

Once H-RPE cells reached confluence, they were treated with tacrolimus (FK506 monohydrate, F4679, Sigma-Aldrich, Poznań, Poland) at concentrations of 0.1, 1, 10, and 100 ng/mL for 6, 12, and 24 h hours. In separate cultures, H-RPE cells were exposed to a combination of 10 ng/mL tacrolimus and 1 µg/mL LPS for 6, 12, and 24 h.

Cell viability was assessed using the 3-(4,5-Dimethylthiazol-2-yl)-2,5-diphenyltetrazolium bromide (MTT) assay, which measures the absorbance of a violet formazan solution. Formazan is generated by the reduction in the yellow tetrazole salt MTT via mitochondrial succinate dehydrogenase in metabolically active cells. The quantity of formazan crystals (water-insoluble) is directly proportional to the number of viable cells in the sample. After 48 h, the MTT assay was performed according to the manufacturer’s instructions. The formazan product was gently resuspended, and absorbance was measured at 580 nm, with a reference wavelength of 720 nm. The percentage of viable cells was determined by comparing the absorbance values of treated samples to those of untreated control cells, which were set at 100% viability.

### 4.3. Isolation of the Total Ribonucleic Acid (RNA)

The extraction of total RNA from tissue samples was initiated using the TRIzol reagent (Invitrogen Life Technologies, Carlsbad, CA, USA; Catalog No. 15596026), following the manufacturer’s protocol with precision. To enhance the purity of the isolated RNA and eliminate residual contaminants, further purification was performed using the RNeasy Mini Kit (QIAGEN, Hilden, Germany; Catalog No. 74104). Additionally, DNase I treatment (Fermentas International Inc., Burlington, ON, Canada; Catalog No. 18047019) was applied to remove any traces of genomic DNA, ensuring high-quality RNA for downstream applications.

The integrity of the extracted RNA was assessed through electrophoresis on a 1% agarose gel containing ethidium bromide (0.5 mg/mL), allowing for visualization and structural evaluation. To determine RNA concentration and purity, absorbance readings were taken at 260 nm, providing essential data on the yield and quality of the isolated RNA.

### 4.4. Messenger RNA and miRNAs Microarray Analysis

A comparative gene expression analysis was conducted to examine TGF-β-related mRNA expression in human retinal pigment epithelial (H-RPE) cells treated with LPS, tacrolimus, or their combination, relative to untreated controls. This analysis utilized the HG-U133_A2 microarray platform (Affymetrix, Santa Clara, CA, USA) with the GeneChip™ 3′ IVT PLUS Reagent Kit (Affymetrix; Cat. No. 902416), following the manufacturer’s protocols and validated methodologies reported in previous studies. A curated list of 86 genes involved in the TGF-β signaling pathway was compiled from the GeneCards human gene database (accessed on 12 March 2025).

The workflow involved the synthesis of double-stranded cDNA using the GeneChip 3′ IVT Express Kit, (Affymetrix, Santa Clara, CA, USA) followed by in vitro transcription for RNA amplification and fragmentation. The resulting amplified RNAs (aRNAs) were hybridized to the microarray, and fluorescence intensity was measured using the Gene Array Scanner 3000 7G, processed with the GeneChip^®^ Command Console^®^ Software. This approach enabled comprehensive profiling of differential gene expression within the TGF-β pathway, offering insights into tacrolimus-mediated molecular alterations.

To further investigate post-transcriptional regulation, miRNA expression profiling was performed using the GeneChip miRNA 2.0 Array (Affymetrix). The procedure followed the manufacturer’s standardized protocol to ensure data quality and reproducibility. Differentially expressed miRNAs between treated and untreated H-RPE cells were analyzed using TargetScan (http://www.targetscan.org/) [63] (accessed on 12 March 2025) and miRanda (http://mirdb.org) [64] (accessed on 12 March 2025), two well-established miRNA target prediction databases.

Predicted miRNA–mRNA interactions with scores above 90 were considered highly reliable and likely to represent functional regulatory relationships. Interactions with scores below 60 were interpreted cautiously and considered candidates for further experimental validation [64,65].

### 4.5. RT-qPCR

In the next phase of molecular analysis, RT-qPCR was performed to validate transcriptional activity changes in genes, as determined from microarray data using the 2^−ΔΔCt^ method. β-actin (ACTB) and Glyceraldehyde-3-Phosphate Dehydrogenase (GAPDH) served as the endogenous reference genes.

The nucleotide sequences of the primers used in RT-qPCR are listed in Table 4. The thermal cycling conditions for RT-qPCR were as follows: reverse-transcription at 45 °C for 10 min, polymerase activation at 95 °C for 2 min, followed by 40 cycles consisting of denaturation at 95 °C for 5 s, annealing at 60 °C for 10 s, and elongation at 72 °C for 5 s.

### 4.6. ELISA Assay

The next phase of the molecular analysis focused on evaluating changes in the levels of selected proteins involved in the TGF-β signaling pathway. This stage was conducted using ELISA, following the manufacturer’s protocol. All ELISA kits were sourced from MyBioSource (San Diego, CA, USA), with the following catalog numbers: Human Mothers Against Decapentaplegic Homolog 4 (Smad4) ELISA Kit (MyBioSource; cat. no. MBS450115), Human Mothers Against Decapentaplegic Homolog 7 (Smad7) ELISA Kit (MyBioSource; cat. no. MBS162196), Transforming Growth Factor Beta-2 (TGF-β2) ELISA Kit (MyBioSource; cat. no. MBS8420054), and Interleukin-2 (IL-2) ELISA Kit (MyBioSource; cat. no. MBS2021996).

### 4.7. Statistical Analysis

The statistical evaluation of the microarray data was conducted using the Transcriptome Analysis Console (Thermo Fisher, Waltham, MA, USA) and Statplus software v. 1.1. These tools were also employed to analyze the results obtained from RT-qPCR and ELISA assays. The initial step in the statistical analysis involved assessing the normality of the data distribution. This was accomplished using the Shapiro–Wilk test, where a *p*-value greater than 0.05 (*p* > 0.05) indicated that the dataset followed a normal distribution, justifying the use of parametric statistical methods. Subsequently, ANOVA (analysis of variance) was performed to identify significant differences in gene expression. When statistical significance was observed (*p* < 0.05), Scheffé’s post hoc test was applied to determine specific differences between groups. The independent samples Student’s *t*-test was used to compare the following groups: H-RPE cells treated with LPS vs. control culture, and H-RPE cells treated with tacrolimus vs. control culture. Gene relationships were analyzed using the STRING database 11.0 (accessed on 3 July 2024). Within STRING, the “strength” parameter (Log10 [observed/expected]) quantifies the enrichment effect, representing the ratio of (1) the number of proteins associated with a given term in the network to (2) the expected number in a randomly generated network of similar size. The false discovery rate (FDR) parameter evaluates enrichment significance, with *p*-values adjusted for multiple testing using the Benjamini–Hochberg procedure [66].

## 5. Conclusions

In conclusion, this study elucidates the molecular mechanisms by which tacrolimus modulates TGF-β signaling, providing a foundation for its potential application in retinal diseases characterized by inflammation and fibrosis. The observed alterations in gene, miRNA, and protein expression profiles suggest that tacrolimus exerts a complex regulatory influence on both canonical and non-canonical pathways, underscoring its potential as an anti-fibrotic and anti-proliferative agent. Further studies, including in vivo validation and clinical trials, are warranted to determine the translational potential of these findings and to explore optimal dosing strategies that maximize therapeutic benefits while minimizing adverse effects.

## Figures and Tables

**Table 1:** Microarray profile of TGF*β*-dependent genes in H-RPE culture exposed to LPS, Tacrolimus, or their combination (|FC| > 4.00 in any comparison).

mRNA	LPS	Tacrolimus	LPS + Tacrolimus		
	log_2_Fold Change				
	H_6 vs. C	H_6 vs. C	H_6 vs. C	H_12 vs. C	H_24 vs. C
*TGF-β2*	+1.27 ± 0.17 *	+1.67 ± 0.65 *	+3.50 ± 0.39 *	+5.14 ± 0.11 *	+5.88 ± 0.27 *
*TGF-β3*	+1.98 ± 0.54 *	+3.45 ± 0.43 *	+5.80 ± 0.41 *	+2.80 ± 0.35 *	+5.10 ± 0.43 *
*TGF-βR1*	−2.13 ± 0.44 *	−3.12 ± 0.76 *	−4.93 ± 0.13 *	−4.06 ± 0.23 *	−5.76 ± 0.44 *
*TGF-βR3*	+2.19 ± 0.31 *	+2.23 ± 0.34 *	+4.39 ± 0.24 *	+4.37 ± 0.30 *	+5.58 ± 0.10 *
*SMAD2*	−1.98 ± 0.57 *	+2.54 ± 0.51 *	+2.62 ± 0.15 *	+2.19 ± 0.46 *	+4.39 ± 0.30 *
*SMAD4*	−2.65 ± 0.81 *	−3.45 ± 0.67 *	−3.21 ± 0.37 *	+5.01 ± 0.28 *	+4.77 ± 0.39 *
*MAPK3*	+2.09 ± 0.41 *	+5.17 ± 0.61 *	+5.12 ± 0.34 *	+3.68 ± 0.31 *	−2.90 ± 0.41 *
*JAK1*	+3.45 ± 0.48 *	+3.21 ± 0.24 *	+3.44 ± 0.20 *	−2.99 ± 0.36 *	+5.45 ± 0.38 *
*JAK2*	−2.34 ± 0.54 *	−3.99 ± 0.81 *	−4.21 ± 0.18 *	−3.51 ± 0.42 *	−2.75 ± 0.37 *
*JAK3*	+2.54 ± 0.18 *	+3.18 ± 0.45 *	+3.98 ± 0.27 *	+4.85 ± 0.22 *	+5.63 ± 0.26 *
*STAT3*	+2.11 ± 0.52 *	+4.77 ± 0.54 *	+5.60 ± 0.40 *	+4.14 ± 0.44 *	−2.85 ± 0.33 *
*EGFR*	−2.45 ± 0.78 *	+4.01 ± 0.62 *	+4.02 ± 0.33 *	−3.55 ± 0.20 *	+5.01 ± 0.37 *
*SMAD7*	−4.56 ± 0.32 *	−2.81 ± 0.81 *	−2.80 ± 0.26 *	+3.98 ± 0.40 *	+5.22 ± 0.29 *
*IFNG*	−2.34 ± 0.38 *	+3.83 ± 0.18 *	+3.91 ± 0.32 *	+2.76 ± 0.45 *	+4.88 ± 0.41 *
*IL-6*	−3.12 ± 0.71 *	+5.13 ± 0.42 *	+5.23 ± 0.28 *	+4.62 ± 0.19 *	−3.17 ± 0.36 *
*CASP3*	+1.65 ± 0.13 *	−2.13 ± 0.18 *	−2.56 ± 0.41 *	+3.11 ± 0.43 *	+4.79 ± 0.32 *
*IL-2*	+1.11 ± 0.44 *	+4.17 ± 0.71 *	+3.69 ± 0.29 *	+5.41 ± 0.23 *	+4.12 ± 0.39 *
*CASP8*	+2.01 ± 0.14 *	+5.16 ± 0.43 *	+5.45 ± 0.31 *	−2.87 ± 0.38 *	+3.91 ± 0.42 *
*KDR*	−3.11 ± 0.43 *	+4.56 ± 0.76 *	+4.92 ± 0.37 *	+5.21 ± 0.21 *	−2.64 ± 0.28 *
*PSAP*	−2.34 ± 0.12	−3.76 ± 0.19 *	−3.33 ± 0.30 *	+4.79 ± 0.24 *	+5.31 ± 0.35 *

Data are presented as mean ± SD; (+), overexpression compared to the control; (−), downregulated compared to the control; C, control culture; H H_6, H_12, H_24, refer to cultures exposed to LPS, tacrolimus, or their combination for 6, 12, and 24 h, respectively; LPS, lipolisaccharide; *TGF-β2*, Transforming Growth Factor Beta 2; *TGF-β3*, Transforming Growth Factor Beta 3; *TGF-βR1*, Transforming Growth Factor Beta Receptor 1; *TGF-βR3*, Transforming Growth Factor Beta Receptor 3; *SMAD2*, Mothers Against Decapentaplegic Homolog 2; *SMAD4*, Mothers Against Decapentaplegic Homolog 4; *MAPK3*, Mitogen-Activated Protein Kinase 3 (ERK1); *JAK1*, Janus Kinase 1; *JAK2*, Janus Kinase 2; *JAK3*, Janus Kinase 3; *STAT3*, Signal Transducer and Activator of Transcription 3; *EGFR*, Epidermal Growth Factor Receptor; *SMAD7*, Mothers Against Decapentaplegic Homolog 7; IFNG, Interferon Gamma; *IL-6*, Interleukin 6; *CASP3*, Caspase 3; *IL-2*, Interleukin 2; *CASP8*, Caspase 8; *KDR*, Kinase Insert Domain Receptor (Vascular Endothelial Growth Factor Receptor 2, VEGFR-2); *PSAP*, Prosaposin; *, a statistically significant difference compared to control (*p* < 0.05).

**Table 2:** Expression profile of miRNAs predicted to regulate selected mRNAs in the TGF-β signaling pathway.

mRNA	miRNA	Target Score	LPS	Tacrolimus	LPS + Tacrolimus		
			H_6 vs. C	H_6 vs. C	H_6 vs. C	H_12 vs. C	H_24 vs. C
*TGF-β-2*	hsa-miR-200a-3p	95	+1.98 ± 0.76 *	+1.65 ± 0.61 *	−2.54 ± 0.19 *	−2.11 ± 0.13 *	−2.19 ± 0.19 *
*SMAD4*	hsa-miR-589-3p	99	−2.12 ± 0.34 *	−2.98 ± 0.61 *	+3.21 ± 0.34 *	+3.22 ± 0.44 *	+2.16 ± 0.78 *
*SMAD7*	hsa-miR-21	92	+1.44 ± 0.17 *	+1.98 ± 0.31 *	+1.19 ± 0.19	+1.87 ± 0.43 *	+2.02 ± 0.45 *
*IL-2*	hsa-miR-27a-5p	94	+2.09 ± 0.65 *	+2.12 ± 0.19 *	−2.13 ± 0.43 *	−2.16 ± 0.22 *	−2.11 ± 0.18 *

Data are presented as mean ± SD; (+), overexpression compared to the control; (−), downregulated compared to the control; C, control culture; H_6, H_12, H_24, refer to cultures exposed to LPS, tacrolimus, or their combination for 6, 12, and 24 h, respectively; TGF-β2, Transforming Growth Factor Beta 2; SMAD4, Mothers Against Decapentaplegic Homolog 4, SMAD7, Mothers Against Decapentaplegic Homolog 7; IL-2, Interleukin 2; *, a statistically significant difference compared to control (*p* < 0.05).

**Table 3:** Changes in the level of selected proteins in cell culture exposed to LPS, and tacrolimus and a control culture.

Protein	Control	LPS	Tacrolimus	LPS + Tacrolimus		
		H_6	H_6	H_6	H_12	H_24
TGF-β2 [pg/mL]	124.11 ± 3.45	328.76 ± 8.91 *	182.34 ± 5.22 *	561.98 ± 9.12 *	701.98 ± 10.11 *	716.11 ± 6.71 *
SMAD4 [ng/mL]	4.67 ± 0.98	6.12 ± 0.44 *	6.89 ± 0.51 *	3.45 ± 0.12 *	3.11 ± 0.18 *	2.99 ± 0.19 *
SMAD7 [ng/mL]	6.19 ± 0.65	5.10 ± 0.35 *	4.58 ± 0.42 *	4.12 ± 0.19 *	4.11 ± 0.76 *	3.45 ± 0.19 *
IL-2 [pg/mL]	176.87 ± 12.65	122.11 ± 9.12 *	98.76 ± 8.45 *	245.17 ± 19.91 *	541.12 ± 12.67 *	671.91 ± 13.45 *

TGF-β2, Transforming Growth Factor Beta 2; SMAD4—Mothers Against Decapentaplegic Homolog 4, SMAD7—Mothers Against Decapentaplegic Homolog 7, IL-2—Interleukin 2; H_6, H_12, H_24, refer to cultures exposed to LPS, tacrolimus, or their combination for 6, 12, and 24 h, respectively; *, a statistically significant difference compared to control (*p* < 0.05).

**Table 4:** Nucleotide sequence of primers.

mRNA	Oligonucleotide Sequence (5′-3′)
*TGF-β2*	Forward: CAGCACACTCGATATGGACCA Reverse: CCTCGGGCTCAGGATAGTCT
*TGF-β3*	Forward: CTGGATTGTGGTTCCATGCA Reverse: TCCCCGAATGCCTCACAT
*TGF-βR1*	Forward: ACTGGCAGCTGTCATTGCTGGACCAG Reverse: CTGAGCCAGAACCTGACGTTGTCATATCA
*TGF-βR3*	Forward: ACCGTGATGGGCATTGCGTTTGCA’ Reverse: GTGCTCTGCGTGCTGCCGATGCTGT
*SMAD2*	Forward: TTGATCGACTGACAGGTGCT Reverse: GACCCCATCCTCACTCCAAA
*SMAD4*	Forward: AAAGGTCTTTGATTTGCGTC Reverse: CTATTCCACCTACTGATCCTG
*SMAD7*	Forward: CAGATTCCCAACTTCTTCTG Reverse: CTCTTGTTGTCCGAATTGAG
*MAPK3*	Forward: AAGATCAGCCCCTTCGAACA Reverse: CCATCAGGTCCTGCACAATG
*JAK1*	Forward: CTGCACCGACTTTGACAACA Reverse: ATCTGCTTCTTGAGGTGGCT
*JAK2*	Forward: AGTAAAAGTCCACCAGCGGA Reverse: AGGAGGGGCGTTGATTTACA
*JAK3*	Forward: CTCTTGGAACTGCTGGAGGA Reverse: AGCAGTGAAGGCATGAGTCT
*STAT3*	Forward: AAAGCAGCAAAGAAGGAGGC Reverse: CTGGCCGACAATACTTTCCG
*EGFR*	Forward: AAAACCGGACTGAAGGAGCT Reverse: TGGATCACACTTTTGGCAGC
*IL-6*	Forward: AGTCCTGATCCAGTTCCTGC CTACATTTGCCGAAGAGCCC
*IL-2*	Forward: CCTCAACTCCTGCCACAATG Reverse: TGTGAGCATCCTGGTGAGTT
*CASP3*	Forward: ACTGGACTGTGGCATTGAGA Reverse: GCACAAAGCGACTGGATGAA
*CASP8*	Forward: GCAAAGGAAGCAAGAACCCA Reverse: CCTGGTGTCTGAAGTTCCCT
*KDR*	Forward: TTACTTGCAGGGGACAGAGG Reverse: TTCCCGGTAGAAGCACTTGT
*PSAP*	Forward: TTGAAAATTGGAGCCTGCCC Reverse: GATCTGTGCGTTCATTCCCC
*GAPDH*	Forward: GGTGAAGGTCGGAGTCAACGGA Reverse: GAGGGATCTCGCTCCTGGAAGA
*ACTB*	Forward: TCACCCACACTGTGCCCATCTACGA Reverse: CAGCGGAACCGCTCATTGCCAATGG

*TGF-β2*, Transforming Growth Factor Beta 2; *TGF-β3*, Transforming Growth Factor Beta 3; *TGF-βR1*, Transforming Growth Factor Beta Receptor 1; *TGF-βR3*, Transforming Growth Factor Beta Receptor 3; *SMAD2*, Mothers Against Decapentaplegic Homolog 2; *SMAD4*, Mothers Against Decapentaplegic Homolog 4; *MAPK3*, Mitogen-Activated Protein Kinase 3 (ERK1); *JAK1*, Janus Kinase 1; *JAK2*, Janus Kinase 2; *JAK3*, Janus Kinase 3; *STAT3*, Signal Transducer and Activator of Transcription 3; *EGFR*, Epidermal Growth Factor Receptor; *SMAD7*, Mothers Against Decapentaplegic Homolog 7; IFNG, Interferon Gamma; *IL-6*, Interleukin 6; CASP3, Caspase 3; *IL-2*, Interleukin 2; *CASP8*, Caspase 8; *KDR*, Kinase Insert Domain Receptor (Vascular Endothelial Growth Factor Receptor 2, VEGFR-2); *PSAP*, Prosaposin.

## Data Availability

The data used to support the findings of this study are included in the article. The data cannot be shared due to third-party rights and commercial confidentiality.

## Abbreviations

The following abbreviations are used in this manuscript:

ACTB	Beta-Actin
AMD	Age-Related Macular Degeneration
ANOVA	Analysis of Variance
C	Control Culture
CASP3/8	Caspase 3/Caspase 8
DR	Diabetic Retinopathy
EGFR	Epidermal Growth Factor Receptor
ELISA	Enzyme-Linked Immunosorbent Assay
EMT	Epithelial–Mesenchymal Transition
FBS	Fetal Bovine Serum
FDR	False Discovery Rate
FGF-B	Fibroblast Growth Factor-Basic
GAPDH	Glyceraldehyde-3-Phosphate Dehydrogenase
H-RPE	Human Retinal Pigment Epithelial (cells)
IL2/6	Interleukin 2/Interleukin 6
IFNG	Interferon Gamma
JAK1/2/3	Janus Kinase 1/2/3
KDR (VEGFR-2)	Kinase Insert Domain Receptor (Vascular Endothelial Growth Factor Receptor 2)
LPS	Lipopolysaccharide
MAPK3	Mitogen-Activated Protein Kinase 3 (ERK1)
miRNA	microRNA
MTT	3-(4,5-Dimethylthiazol-2-yl)-2,5-diphenyltetrazolium bromide
NFAT	Nuclear Factor of Activated T Cells
PCR	Polymerase Chain Reaction
PPI	Protein–Protein Interaction
PVR	Proliferative Vitreoretinopathy
PSAP	Prosaposin
RNA	Ribonucleic Acid
RT-qPCR	Quantitative Reverse-Transcription Polymerase Chain Reaction
SMAD2/4/7	Mothers Against Decapentaplegic Homolog 2/4/7
STAT3	Signal Transducer and Activator of Transcription 3
STRING	Search Tool for the Retrieval of Interacting Genes/Proteins
TGF-β1/2/3	Transforming Growth Factor Beta 1/2/3
TGF-βR1/3	Transforming Growth Factor Beta Receptor 1/3
VEGFR-2	Vascular Endothelial Growth Factor Receptor 2

## Appendix A

**Table A1:** Student’s *t*-test results for RT-qPCR-based differential gene expression in H-RPE cells treated with LPS or tacrolimus in comparison to untreated cells (control).

mRNA	LPS vc. C	Tacrolimus vs. C
*TGF-β2*	NS	*p* = 0.043
*TGF-β3*	*p* = 0.021	*p* = 0.012
*TGF-βR1*	*p* = 0.041	*p* = 0.013
*TGF-βR3*	*p* = 0.003	*p* = 0.011
*SMAD2*	NS	*p* = 0.025
*SMAD4*	*p* = 0.009	*p* = 0.028
*SMAD7*	*p* = 0.001	*p* = 0.029
*JAK1*	*p* = 0.019	*p* = 0.013
*JAK2*	*p* = 0.041	*p* = 0.027
*JAK3*	*p* = 0.039	*p* = 0.021
*STAT3*	*p* = 0.047	*p* = 0.001
*EGFR*	*p* = 0.023	*p* = 0.017
*MAPK3*	*p* = 0.041	*p* = 0.001
*IFNG*	*p* = 0.034	*p* = 0.031
*IL-6*	*p* = 0.002	*p* = 0.012
*CASP3*	*p* = 0.049	*p* = 0.043
*CASP8*	*p* = 0.047	*p* = 0.013
*IL-2*	NS	*p* = 0.014
*KDR*	*p* = 0.022	*p* = 0.019
*PSAP*	*p* = 0.039	*p* = 0.038

C, control culture; NS, not significant (*p* ≥ 0.05); *TGF-β2*, Transforming Growth Factor Beta 2; *TGF-β3*, Transforming Growth Factor Beta 3; *TGF-βR1*, Transforming Growth Factor Beta Receptor 1; *TGF-βR3*, Transforming Growth Factor Beta Receptor 3; *SMAD2*, Mothers Against Decapentaplegic Homolog 2; *SMAD4*, Mothers Against Decapentaplegic Homolog 4; *MAPK3*, Mitogen-Activated Protein Kinase 3 (ERK1); *JAK1*, Janus Kinase 1; *JAK2*, Janus Kinase 2; *JAK3*, Janus Kinase 3; *STAT3*, Signal Transducer and Activator of Transcription 3; *EGFR*, Epidermal Growth Factor Receptor; *SMAD7*, Mothers Against Decapentaplegic Homolog 7; *IFNG*, Interferon Gamma; *IL-6*, Interleukin 6; *CASP3*, Caspase 3; *IL-2*, Interleukin 2; *CASP8*, Caspase 8; *KDR*, Kinase Insert Domain Receptor (Vascular Endothelial Growth Factor Receptor 2, VEGFR-2); *PSAP*, Prosaposin.

**Table A2:** One-way ANOVA and Scheffé’s post hoc test results for RT-qPCR-based differential gene expression in H-RPE cells treated with LPS and tacrolimus.

mRNA	ANOVA *p*-Value	Scheffé’s Post Hoc Test Results		
		H_6 vs. C	H_12 vs. C	H_24 vs. C
*TGF-β2*	0.002	*p* = 0.021	*p* = 0.008	*p* = 0.016
*TGF-β3*	0.005	NS	*p* = 0.033	*p* = 0.009
*TGF-βR1*	0.001	*p* = 0.006	*p* = 0.018	*p* = 0.041
*TGF-βR3*	0.010	*p* = 0.028	*p* = 0.019	NS
*SMAD2*	0.012	*p* = 0.036	*p* = 0.031	*p* = 0.007
*SMAD4*	0.020	NS	*p* = 0.044	*p* = 0.009
*SMAD7*	0.003	*p* = 0.022	*p* = 0.005	*p* = 0.019
*JAK1*	0.008	*p* = 0.017	*p* = 0.011	*p* = 0.038
*JAK2*	0.018	*p* = 0.031	*p* = 0.006	*p* = 0.045
*JAK3*	0.035	NS	*p* = 0.027	*p* = 0.033
*STAT3*	0.002	*p* = 0.015	*p* = 0.009	*p* = 0.007
*EGFR*	0.009	*p* = 0.023	*p* = 0.032	*p* = 0.041
*MAPK3*	0.006	*p* = 0.037	*p* = 0.004	*p* = 0.033
*IFNG*	0.004	*p* = 0.029	*p* = 0.007	*p* = 0.015
*IL6*	0.005	*p* = 0.034	*p* = 0.016	*p* = 0.008
*CASP3*	0.001	*p* = 0.022	*p* = 0.009	*p* = 0.005
*CASP8*	0.004	*p* = 0.033	*p* = 0.021	*p* = 0.008
*IL-2*	0.031	NS	*p* = 0.044	NS
*KDR*	0.014	*p* = 0.008	NS	NS
*PSAP*	0.003	*p* = 0.025	*p* = 0.038	*p* = 0.012

C, control culture; H_6, H_12, H_24, culture exposed to tacrolimus for 6, 12, 24 h; NS, not significant (*p* ≥ 0.05); *TGF-β2*, Transforming Growth Factor Beta 2; *TGF-β3*, Transforming Growth Factor Beta 3; *TGF-βR1*, Transforming Growth Factor Beta Receptor 1; *TGF-βR3*, Transforming Growth Factor Beta Receptor 3; *SMAD2*, Mothers Against Decapentaplegic Homolog 2; *SMAD4*, Mothers Against Decapentaplegic Homolog 4; *MAPK3*, Mitogen-Activated Protein Kinase 3 (ERK1); *JAK1*, Janus Kinase 1; *JAK2*, Janus Kinase 2; *JAK3*, Janus Kinase 3; *STAT3*, Signal Transducer and Activator of Transcription 3; *EGFR*, Epidermal Growth Factor Receptor; *SMAD7*, Mothers Against Decapentaplegic Homolog 7; *IFNG*, Interferon Gamma; *IL-6*, Interleukin 6; *CASP3*, Caspase 3; *IL-2*, Interleukin 2; *CASP8*, Caspase 8; *KDR*, Kinase Insert Domain Receptor (Vascular Endothelial Growth Factor Receptor 2, VEGFR-2); *PSAP*, Prosaposin.
